# Use of risk stratification to target therapies in patients with recent onset arthritis; design of a prospective randomized multicenter controlled trial

**DOI:** 10.1186/1471-2474-10-71

**Published:** 2009-06-18

**Authors:** Susanne JJ Claessen, Johanna MW Hazes, Margriet AM Huisman, Derkjen van Zeben, Jolanda J Luime, Angelique EAM Weel

**Affiliations:** 1 Department of Rheumatology, Erasmus MC, University Medical Center, Dr Molewaterplein, Rotterdam, The Netherlands; 2 Department of Rheumatology, Sint Franciscus Gasthuis, Kleiweg, Rotterdam, The Netherlands; 3 Department of Rheumatology, Maasstad Ziekenhuis, Olympiaweg, Rotterdam, The Netherlands

## Abstract

**Background:**

Early and intensive treatment is important to inducing remission and preventing joint damage in patients with rheumatoid arthritis. While intensive combination therapy (Disease Modifying Anti-rheumatic Drugs and/or biologicals) is the most effective, rheumatologists in daily clinical practice prefer to start with monotherapy methotrexate and bridging corticosteroids. Intensive treatment should be started as soon as the first symptoms manifest, but at this early stage, ACR criteria may not be fulfilled, and there is a danger of over-treatment. We will therefore determine which induction therapy is most effective in the very early stage of persistent arthritis. To overcome over-treatment and under-treatment, the intensity of induction therapy will be based on a prediction model that predicts patients' propensity for persistent arthritis.

**Methods:**

A multicenter stratified randomized single-blind controlled trial is currently being performed in patients 18 years or older with recent-onset arthritis. Eight hundred ten patients are being stratified according to the likelihood of their developing persistent arthritis. In patients with a high probability of persistent arthritis, we will study combination Disease Modifying Antirheumatic Drug therapy compared to monotherapy methotrexate. In patients with an intermediate probability of persistent arthritis, we will study Disease Modifying Antirheumatic Drug of various intensities. In patients with a low probability, we will study non-steroidal anti-inflammatory drugs, hydroxychloroquine and a single dose of corticosteroids. If disease activity is not sufficiently reduced, treatment will be adjusted according to a step-up protocol. If remission is achieved for at least six months, medication will be tapered off. Patients will be followed up every three months over two years.

**Discussion:**

This is the first rheumatological study to base treatment in early arthritis on a prediction rule. Treatment will be stratified according to the probability of persistent arthritis, and different combinations of treatment per stratum will be evaluated. Treatment will be started early, and patients will not need to meet the ACR-criteria for rheumatoid arthritis.

**Trial registration:**

This trial has been registered in Current Controlled Trials with the ISRCTN26791028.

## Background

Rheumatoid arthritis (RA) is a chronic inflammation of the joints that causes structural joint damage, affecting daily physical functioning and often limiting social functioning and work participation. [1-7] Its prevalence in Europe is around 1%.[8] Although its primary cause is unknown, multiple genetic and environment factors seem to be involved.[9] While no cure has been found, combinations of disease-modifying anti-rheumatic drugs (DMARDs) and biologicals have proved very effective in reducing disease activity and radiological progression, especially when given early in the disease course. [10-22]

The studies to have reported this were all performed in patients who fulfilled the ACR criteria of RA. But if the treatment of RA is to be improved, it is necessary to go beyond the ACR criteria, a number of which apply to features of later stages of the disease, and are thus insensitive in early rheumatoid arthritis.[23]

The need for earlier treatment is recognized by the EULAR, who recommend not only that "patients at risk of developing persistent or erosive arthritis should be started with DMARDs as early as possible, even if they do not yet fulfil established classification criteria for inflammatory rheumatological diseases," but also "among the DMARDS, methotrexate is considered to be the anchor drug, and should be used first in patients at risk of developing persistent disease."[24] However, it is unclear which criteria should be used for the early identification of persistent disease.

One way of identifying patients at risk for persistent disease would be by using early arthritis-prediction models[25,26], none of which have yet been used for treatment decisions in daily practice. Visser et al developed a prediction rule with seven factors: 1.) symptom duration at the first visit, 2.) morning stiffness for at least one hour, 3.) arthritis in three or more joints, 4.) bilateral compression pain in the MTP joints, 5.) IgM-RF positivity, 6.) anti-CCP positivity, and 7.) erosions on radiographs of the hands or feet. The odds ratio (OR) of each variable was simplified by substituting the ORs with weighted scores from 0 to 13 points. The range of probabilities to which this translated lay between a 10% probability of developing persistent arthritis when one point was scored, and a 99% probability when 13 points were scored. Greater detail is available in Visser et al.[26]

The main aim of the present study is to determine which prediction-guided treatment strategy is superior in patients with recent-onset arthritis who, on the basis of the Visser Prediction rule, are stratified as having a low, intermediate or high risk of developing persistent arthritis.

## Methods

### Study design

The Treatment in the Rotterdam Early Arthritis Cohort (tREACH) is a multicenter stratified randomized single-blind controlled trial. Patients aged ≥ 18 years with at least one arthritis (symptom duration less than one year) will be invited to participate. After providing written informed consent, eligible patients will be stratified into three groups according to the likelihood (on the basis of the prediction model of Visser described previously) of their progressing to persistent arthritis. Patients in each stratum will be randomized to three treatment strategies (a so-called 3 × 3 design). Variable block randomization will be accomplished by a coordination center by telephone during working hours. Treatment strategy will be predefined for the first year, after which the rheumatologist will be free to prescribe as they see fit. Follow-up will take place every three months over a period of two years.

### Recruitment

Patients are currently being recruited from a large prospective cohort study, the Rotterdam Early Arthritis Cohort (REACH study). This includes patients aged ≥ 16 years who live in the area of Rotterdam and have inflammatory joint complaints whose symptoms they have had for less than one year. The REACH study is being conducted in cooperation with the general practitioners in the region.[3,5]

To be included in the present study, patients should have had at least one arthritis confirmed by a rheumatologist. They will be excluded 1.) if they are were diagnosed with a crystal arthropathy, infectious or post-infectious arthritis, or an autoimmune rheumatic disorder other than RA; 2.) if they have been on DMARD therapy, corticosteroids within the last three months, or on concomitant treatment with any experimental drug; or 3.) if there are any of the following: contra-indications for the initial study medication (history of chronic liver disease; excessive alcohol and drug use; pregnancy or the desire to become pregnant during the study; childbearing potential without adequate contraception; or the following laboratory abnormalities: leucopenia (< 3.0 × 10^9^/l), thrombocytopenia (< 150 × 10^9^/l), ASAT/ALAT > double the upper normal value creatinine level > 150 μmol/l).

### Interventions

The medical interventions evaluated in tREACH include three conventional DMARDs: methotrexate (MTX), sulfasalazine (SSZ) and hydroxychloroquine (HCQ). Since intensive treatment is important early in the disease course, this trial is designed to produce the greatest treatment differences during the first three months of therapy (induction therapy).

In this 3 × 3 design, three treatment strategies will be evaluated per probability stratum:

1. A high-probability group (HP), in which the risk of developing persistent arthritis is >70%

2. An intermediate-probability group (IP), in which this risk lies between 30% and 70%

3. A low-probability group (LP), in which this risk is < 30%

Treatment strategies will be tightly controlled, with patients being examined every three months.[27] The aim of treatment is to reduce disease activity as expressed by the Disease Activity Score (DAS) ≤ 2.4.[28] If initial treatment fails (DAS>2.4), the treatment will be intensified using the next step of the prescribed treatment strategy. If the DAS ≤ 2.4, initial treatment will be continued; if the patient meets the criteria for remission (DAS ≤ 1.6) over three successive visits, it will be tapered off. If there is a discrepancy between the DAS score and the opinion of the rheumatologist, an additional DAS will be performed two weeks later. If the discrepancy persists, the initial DAS will be used.

### Induction therapy

**Figure 1:**
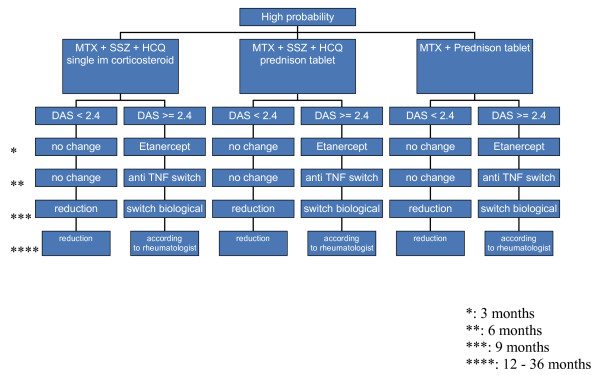
**Medication strategies in the high-probability group**. Dosages: MTX: 25 mg/week oral or subcutaneously. SSZ: 2 grams/day. HCQ: 200 mg twice a day. Single-dose corticosteroids intramuscularly: kenacort 80 mg once intramuscularly, or depomedrol 120 mg once intramuscularly. Prednisone scheme: Week 1–4 15 mg a day. Week 5–6: 10 mg a day. Week 7–8: 5 mg a day. Week: 9–10: 2.5 mg a day. Etanercept: 50 mg once a week subcutaneously. Adalumimab: 40 mg once every two weeks subcutaneously. Rituximab: 1 gram intravenously, repeated after two weeks. Leflunomide: 20 mg once daily.

**Figure 2:**
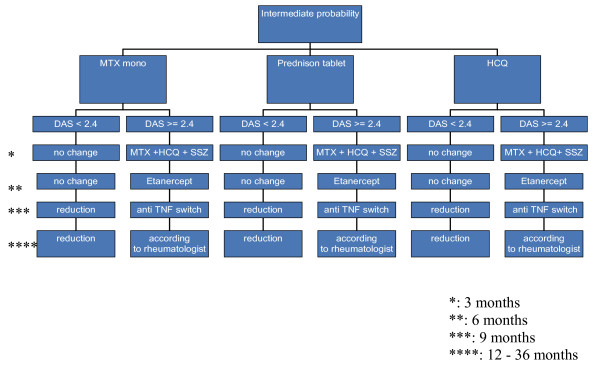
**Medication strategies in the intermediate probability group**. Dosages: see figure 1 legend.

**Figure 3:**
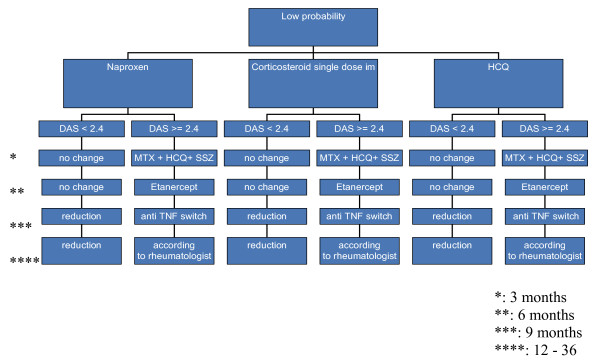
**Medication strategies in the low-probability group**. Dosages: see figure 1 legend. NSAID: Naproxen 1000 mg/day.

Figures 1, 2 and 3 show the the types, dosages of induction and step-up therapy for the three probability groups.

In the HP group, the three induction therapies are:

• Combination DMARD (MTX, SSZ and HCQ) therapy with single-dose corticosteroids intramuscularly

• Combination DMARD (MTX, SSZ and HCQ) therapy with cumulative high-dose corticosteroids

• MTX with cumulative high-dose corticosteroids

In the high probability group, osteoporosis prophylaxis (risedronate 35 mg once a week; combination of calcium and vitamin D 500/440) will be given to patients who are treated with cumulative high-dose corticosteroids during induction therapy.

In the IP group, the three induction therapies are:

• MTX

• Cumulative high-dose corticosteroids

• HCQ

In the LP group, the three induction therapies are:

• Naproxen

• Single-dose corticosteroids intramuscularly

• HCQ

### Treatment failure (DAS > 2.4)

If DAS>2.4, the DAS will be measured every three months, and treatment will be intensified by step-up therapy according to the next step of the treatment protocol (figure 1, 2 and 3).

For patients in the HP group step-up therapy will be the combination of MTX and anti-TNF α (Etanercept), if therapy still fails after 3 months patients will be switched to another anti-TNF α (Adalumimab). [29-31] The final step-up will be B-cell depletion (Rituximab).

The initial step-up in the IP group and LP group will be the combination DMARDs (MTX-SSZ-HCQ). If failure persists treatment will be intensified to the combination of MTX and anti-TNF α (Etanercept), and finally to another anti-TNF α (Adalumimab).

### Disease remission (DAS ≤ 1.6)

In all probability, if remission (DAS ≤ 1.6) is achieved over three consecutive visits, a group's medication will be tapered off. Step-down of therapy in patients using the combination of MTX-SSZ-HCQ will start with tapering SSZ in the first three months, followed, if this is successful, by tapering off MTX over three months and finally HCQ will be stopped. If patients using the combination of MTX and anti-TNF α achieve remission, first the anti-TNF will be stopped and, if remission sustained, MTX is finally stopped. Patients achieving remission during B-cell depletion therapy, first of all B-cell depletion will be stopped and if this is successful, MTX will be tapered off.

### Drug side-effects and concomitant drugs

In the event of side effects or contra-indications for the study medication, patients will be treated with leflunomide 20 mg 1 dd. During follow-up, Non-Steroidal Anti-Inflammatory Drugs (NSAIDs) and intra-articular injections will be allowed. Safety monitoring of treatment will take place according to Dutch guidelines.

### Data collection

The primary outcome parameters are functional ability (HAQ), disease activity (DAS), and radiological joint damage (the modified Sharp van der Heijde score (modified SvH)).

The HAQ is a self-report functional status measure assessing eight activities 1.) dressing and grooming, 2.) arising, 3.) eating, 4.) walking, 5.) hygiene, 6.) reaching, 7.) gripping, and 8.) common daily activities.[32] For each of these categories, patients report the amount of difficulty they have performing two or three specific activities. The HAQ score is 0–3 with a minimal clinically important difference of 0.22.

The DAS includes the Ritchie articular index, the 44 swollen joint count, the Erythrocyte Sedimentation Rate, and a general health assessment on a VAS.[28] The DAS ranges between 0 and 10.

The modified SvH score will be used to determine the extent of joint damage.[33] Single-emulsion radiographs of the hands (posteroanterior view) and feet (anteroposterior view) will be obtained and digitized for blinded reading. Two independent blinded readers will score the radiographs of each patient by assessing joint erosions (0–5 scale) and joint-space narrowing (0–4 scale) per joint.

The HAQ and DAS will be scored every three months, and X-ray data will be collected every six months over two years of follow-up.

Secondary outcome parameters will include 1.) WHO/ILAR and EULAR Core-set measurements for clinical trials [34,35], 2.) self-assessed disease activity (RADAI)[36], and 3.) Quality of life on the basis of the SF-36 and the Euroqol [37,38].

Using self-reported questionnaires and chart data, additional data will be collected on direct and indirect healthcare costs, and adverse and seriously adverse events.

### Data monitoring and ethical approval

This study is being monitored by a steering committee. To verify the data, the study coordinator, a member of the steering committee, will visit the participating centers frequently. All patient data collected during the study will be analyzed and documented according to Good Clinical Practice.

Ethical approval has been obtained from the Ethics Committees at Erasmus Medical Center, Sint Franciscus Gasthuis, Maasstad Hospital, Vlietland Hospital, Albert Schweitzer Hospital, The Oosterschelde Hospital, Hospital Walcheren, and ZorgSaam Hospital.

## Statistics

### Sample size

Sample-size calculations were based on the AUC HAQ using primary data from the BeST study[15], where the mean AUC HAQ of the combination therapy was 7.7 (SD 5.5) and the mean AUC HAQ of monotherapy was 10.5 (SD 7.4)

To detect this difference using a significance level of α = 0.05 and a power of 80%, ninety patients are needed in each treatment arm. This number of patients is sufficient to detect a difference of 6.1 AUC DAS and 20% of patients with or without radiographic progression towards baseline (α = 0.05; power 80%).

In total, 810 patients will be needed. Allowing for a participation rate of 80%, 1012 patients will have to be screened, which corresponds with 337 patients in each probability group. With seven hospitals involved, we expect to recruit 34 eligible patients a month.

### Analysis

Baseline characteristics will be presented with descriptive statistics. Number, mean, standard deviation, and minimum and maximum values will be used for continuous data; and absolute and relative frequencies will be used for categorical data.

Data on the primary outcomes HAQ and DAS will be analyzed per strata of probability (HP, IP and LP) over time using the AUC (area under the curve), which is an appropriate method for detecting therapy difference early in the follow-up period. Analysis will be performed using intention-to-treat analysis and per-protocol analysis. Data on radiological progression will be analyzed using the non-parametric Mann-Whitney U test. All data will be analyzed using STATA 9.0 and R.

### Cost-effectiveness analysis

In the study, direct and indirect costs will all be considered within the one-year period following randomization. Direct medical costs will be calculated on the basis of use of resources within the healthcare sector. Indirect costs are defined as the cost of production lost through illness or its treatment.

Medication use will be recorded on the basis of the trial medication prescribed and will be valued on the basis of national cost prices excluding value added tax (VAT) and markups for the acquisition and administration of medication. The use of over-the-counter medication will be recorded through patient questionnaires. The cost of over-the-counter medications will be calculated on the basis of retail prices.

Adverse events and serious adverse events will be recorded during the trial period. To estimate the average cost per type of adverse event, scenario studies will be conducted.

Data on medical care utilization outside the hospital and on absence from work will be collected using a patient questionnaire that includes questions on healthcare visits, medication, quality of life, and number of working days lost due to joint complaints or other disorders. As a basis for establishing the level of production losses, we will use the average income per person by age and gender for 2008–2010.

Cost data will be analyzed per probability stratum by comparing the three treatment groups in each stratum. Incremental cost-effectiveness ratios will be calculated by dividing the difference between the costs of the treatment strategies by the difference in the DAS and HAQ. At baseline, 6 months and 12 months, the effects of arthritis and therapy on utility will be measured using the EQ5d.

## Discussion

The overall objective of this study is to determine the most effective induction therapy for patients with early arthritis who are at risk of persistent arthritis. An important difference between this study and previous studies is that we are able to start treatment before patients have fulfilled the ACR criteria. To do so, we are being guided by the prediction model of Visser for early arthritis, which is used to stratify patients according to a low, intermediate or high risk of persistent arthritis. The induction therapies we use reflect current rheumatology practice, ranging from NSAIDs in the low probability group to combination DMARD therapy in the high probability group.

This study has two specific objectives focused on induction therapy in the HP group. The first is to establish whether MTX-monotherapy is as effective as combination DMARD therapy (MTX-SSZ-HCQ) in patients who are at a high risk of persistent arthritis. For although established RA combination therapy have been shown to perform better than monotherapy in terms of reducing disease activity and radiological progression [10-15,17,19,20], there is no evidence in early arthritis that monotherapy MTX is as effective as combination DMARD therapy, despite its accepted use in daily clinical practice. Moreover all these studies analyzing the combination of DMARDs show that their effectiveness may have been modified by corticosteroids.

Therefore the second specific objective concerns the use of corticosteroids. In daily practice corticosteroids are widely used as bridging therapy since the maximum therapeutic effect of DMARD will take at least three months. But until now, it is unclear what the starting dosage should be and for how long corticosteroids should be prescribed. We intend to establish whether one intramuscular bolus of prednisolone is as effective as the cumulative high dose over three months of oral prednisolone.

Our choice of treatment in the intermediate group was based on the 50% chance that people in this group have a self-limiting disease, which makes overtreatment an important issue. To be able to choose the optimal benefit-harm ratio for these patients, we decided to include various treatment intensities, MTX being the most intensive. We expect single DMARD therapy to be safe, and that overtreatment will probably be less harmful than the risk of irreversible joint damage [39]. Furthermore, if any of these patients goes into remission, their medication will also be tapered off quickly.

Mild treatment options were chosen for patients in the low probability group, hardly any of whom are expected to progress to persistent arthritis.

The trial is being conducted in the southwestern Netherlands, and has been received with great enthusiasm by the participating centers, particularly because rheumatologists there feel that it will answer very practical questions. Because treatment choices have been made by consensus between all participating rheumatologists, it is likely that the results of the trial will be implemented easily in daily practice.

## Competing interests

The authors declare that they have no competing interests.

## Authors' contributions

The study was conceived by JMWH and AEAMW, with JMWH, MAMH, DZ and AEAMW participating in its design. The trial is being coordinated by AEAMW. The manuscript was prepared by SJJC, JJL and AEAMW. The final draft was read and approved by all authors.

## Pre-publication history

The pre-publication history for this paper can be accessed here:


